# Supplementary material for: Neural Sequences Underlying Directed Turning in C. elegans
Source: bioRxiv. 2024 Aug 11:2024.08.11.607076. Preprint. [Version 1] doi: 10.1101/2024.08.11.607076 (PMC11326294; doi:10.1101/2024.08.11.607076)
Supplement: Supplement 1 [file NIHPP2024.08.11.607076v1-supplement-1.pdf]

bioRxiv preprint doi: https://doi.org/10.1101/2024.08.11.607076; this version posted August 11, 2024. The copyright holder for this preprint (which was not certified by peer review) is the author/funder, who has granted bioRxiv a license to display the preprint in perpetuity. It is made available under aCC-BY 4.0 International license.

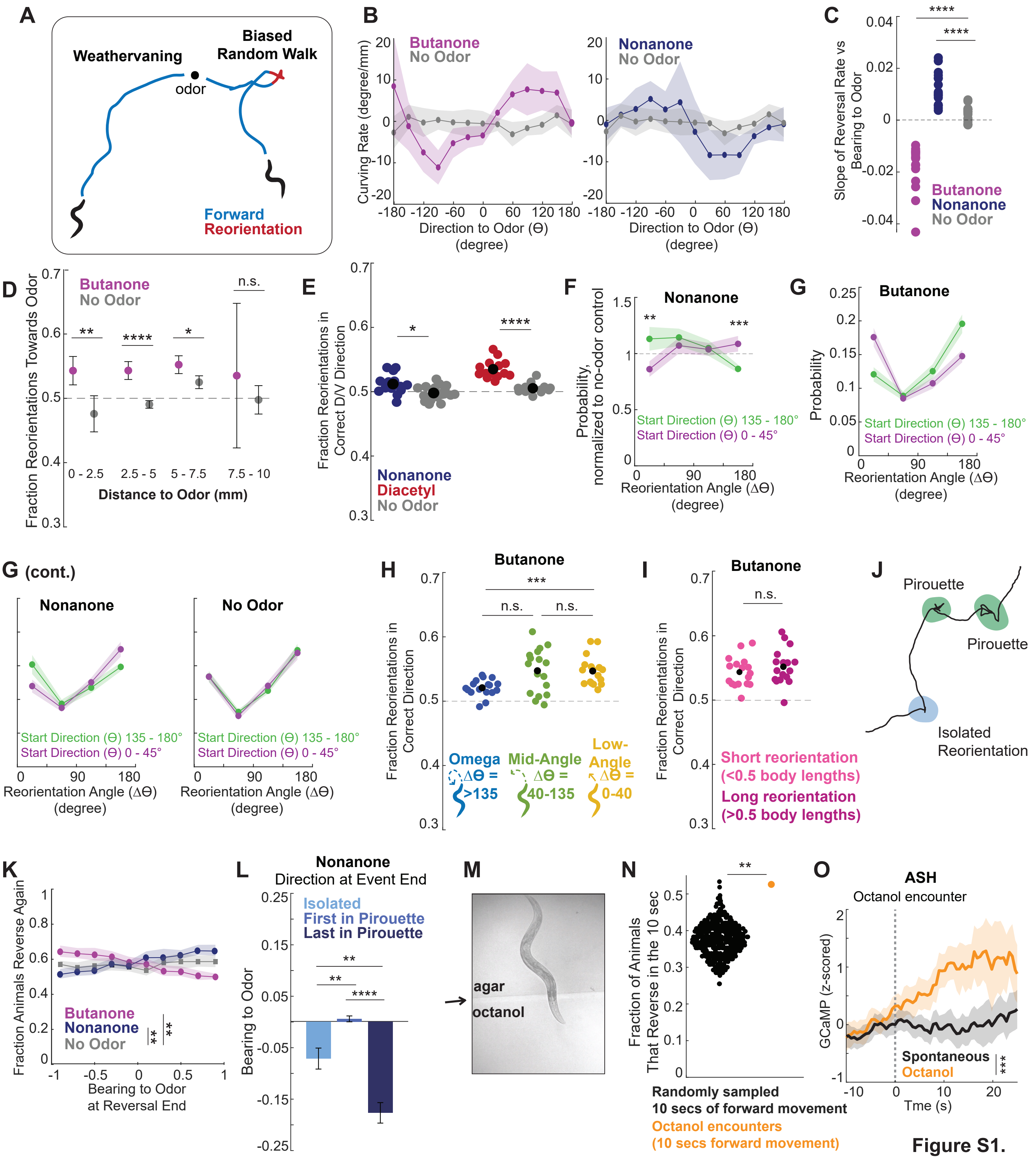

Figure S1.

bioRxiv preprint doi: https://doi.org/10.1101/2024.08.11.607076; this version posted August 11, 2024. The copyright holder for this preprint (which was not certified by peer review) is the author/funder, who has granted bioRxiv a license to display the preprint in perpetuity. It is made available under aCC-BY 4.0 International license.

Supplemental Figure 1, Related to Fig. 1

A) Visualization of the two main chemotaxis strategies: weathervaning (left) and biased random walk (right). Animals weathervane by bending their direction of forward movement in a favorable direction, either towards the attractive odor or away from the aversive odor[38]. Animals execute a biased random walk by increasing the likelihood of initiating reorientations (red) when they are moving in an unfavorable direction in the odor gradient (forward movement shown in blue)[19].

B) Weathervaning behavior in olfactory gradients. To test for the presence of weathervaning, we examined the curving rate of forward movement when animals had different directions to the odor (Θ). Curving rate is the change in the animal's heading divided by their change in displacement over 1 second, which can be thought of as a measure of how much and which way animals are bending forward runs (see Methods for further details). As previously shown[38], animals bend runs towards attractive odors (butanone, sign of Θ is the same as the sign of the curving rate). We further saw weaker evidence that animals weathervane away from aversive odors (nonanone, sign of the curving is the opposite of the sign of Θ). Data is mean ± 95% CI

C) Visualization of statistics from Fig. 1B, showing the slopes of fitting a linear fit to each recordings' reversal rate vs bearing to odor plot. ****p<.0001, Wilcoxon's Rank Sum Test with Bonferroni Correction comparing slopes of the reversal rate. n = 16-18 recording plates with 20-100 animals each. Each dot is one recording.

D) Fraction of reorientations that turn the animal towards the odor across different regions of the plate (distance to odor is indicated below, plates are 10 mm wide). Very few animals navigate away from the butanone, which results in the large error bars on the 7.5-10mm from odor bin. ****p<.0001, Wilcoxon's Rank Sum Test with Bonferroni Correction. n = 16-18 recording plates with 20-100 animals each. Each dot is the mean of all recorded plates, error bars show ± 95% CI.

E) For wild type animals, fraction of reorientations that turn the animal in the correct dorsal or ventral direction, comparing nonanone to no odor and diacetyl to no odor (with odor plates were recorded on different days, so each has their own no odor control). ****p<.0001, Wilcoxon's Rank Sum Test with Bonferroni Correction. n = 12-18 recordings. Black dot shows data mean.

F) Change in direction ($\Delta\Theta$) executed by animals that start with a large or small angle to the odor (Θ). As animals naturally tend to execute turns of certain angles, the data is normalized to no odor controls for ease of visualization. Note that the "goal" for nonanone is the opposite of the goal turn for butanone – animals that begin facing towards odor (purple) are best served by executing larger angle turns to turn away from the odor, while animals that begin facing away from the odor (green) are best served by executing small angle turns. We indeed see such a behavior modulation. ****p<.0001, Wilcoxon's Rank Sum Test with Bonferroni Correction. n = 16-18 recording plates. Data show mean ± 95% CI.

G) Non-normalized version of the data shown in Fig. 1I and S1F. Change in direction ($\Delta\Theta$) executed by animals that start with a large or small angle initial direction to the odor (Θ). We chose to normalize the data due to the distinctive V shape shown here both with or

bioRxiv preprint doi: https://doi.org/10.1101/2024.08.11.607076; this version posted August 11, 2024. The copyright holder for this preprint (which was not certified by peer review) is the author/funder, who has granted bioRxiv a license to display the preprint in perpetuity. It is made available under aCC-BY 4.0 International license.

without odor. This characteristic shape indicates that, in general, *C. elegans* are more likely to execute small angle reorientations (0-45) or larger angle (90-180), but are less likely to do 45-90 degree turns. To emphasize the change in their behavior due to the presence of an odor, rather than this natural tendency, we normalized the rate in the presence of an odor to the rate of reversals of the same angle ($\Delta\Theta$) in no-odor control videos recorded in parallel.

H) Fraction of reorientations that turn the animal in the correct dorsal or ventral direction during butanone chemotaxis, split by the type of reorientation. Omega reorientations end with a distinctive high angle turn >135 degrees[47] and have a characteristic body shape (see details in Methods), mid-angle reorientations end with a turn between 40-135 degrees, and low-angle reversals have a turn of 0-40 degrees. ****p<.0001, Wilcoxon's Rank Sum Test with Bonferroni Correction. n = 17 recording plates. Black dots show data mean.

I) Fraction of reorientations that turn the animal in the correct dorsal or ventral direction among low angle reversals, split by reorientation length. Short reversals are less than 0.5 body lengths. ****p<.0001, Wilcoxon's Rank Sum Test. n = 17 v. Black dots show data mean.

J) Example animal movement path during chemotaxis showing a single, isolated reorientation (blue) and two examples of repeated reorientations that form a pirouette (green).

K) Fraction of animals that reverse in the next 13 seconds depending on their bearing to the odor at the end of their previous reversal. Animals that end an individual reversal in an unfavorable direction (away from butanone or towards nonanone) are more likely to reverse again. ****p<.0001, Wilcoxon's Rank Sum Test with Bonferroni Correction comparing slopes of the reversal rate. n = 16-18 recording plates. Data are mean ± 95% CI.

L) Bearing to odor at the end of isolated reorientations, the first reorientation of a pirouette, or the last reorientation of a pirouette for animals in a nonanone gradient. Pirouettes are defined as clusters of consecutive reorientations separated by less than 13 seconds. ****p<.0001, Wilcoxon's Rank Sum Test with Bonferroni Correction. n = 16 recording plates. Data are mean ± SEM.

M) Example animal encountering octanol during whole brain calcium imaging, showing that the difference between the baseline and octanol agars is identifiable by eye. The agar boundary is indicated with a black arrow to the left of the image. Octanol encounters are scored by hand.

N) Fraction of animals that start a reversal in a 10 second interval. "Octanol" specifically looks at whether animals start a reversal in the 10 seconds following an octanol encounter. "Spontaneous" is from data looking at if animals reverse in a randomly chosen 10 second interval of spontaneous movement on baseline agar (not octanol). This fraction is calculated by looking at the fraction of animals that reverse in a fixed number of random intervals, which is chosen based on the number of intervals where the animal was on octanol (n = 132). Each dot shows one random sample of data. This process was then repeated 500 times to generate the distribution in black. Statistics compare this

bioRxiv preprint doi: https://doi.org/10.1101/2024.08.11.607076; this version posted August 11, 2024. The copyright holder for this preprint (which was not certified by peer review) is the author/funder, who has granted bioRxiv a license to display the preprint in perpetuity. It is made available under aCC-BY 4.0 International license.

distribution to the actual fraction of animals reversing on octanol in a one tailed test. The octanol value was at the 99th percentile of the dataset. **p<.01

O) ASH activity as animals encounter the aversive octanol barrier is shown in orange, showing increasing aversive sensory drive. Black line shows ASH activity during similar epochs of spontaneous forward movement. Gray dashed line shows octanol encounter. ****p<.0001, Wilcoxon's Rank Sum Test comparing average activity on octanol and during spontaneous movement post octanol encounter, data are mean ± 95% CI.

bioRxiv preprint doi: https://doi.org/10.1101/2024.08.11.607076; this version posted August 11, 2024. The copyright holder for this preprint (which was not certified by peer review) is the author/funder, who has granted bioRxiv a license to display the preprint in perpetuity. It is made available under aCC-BY 4.0 International license.

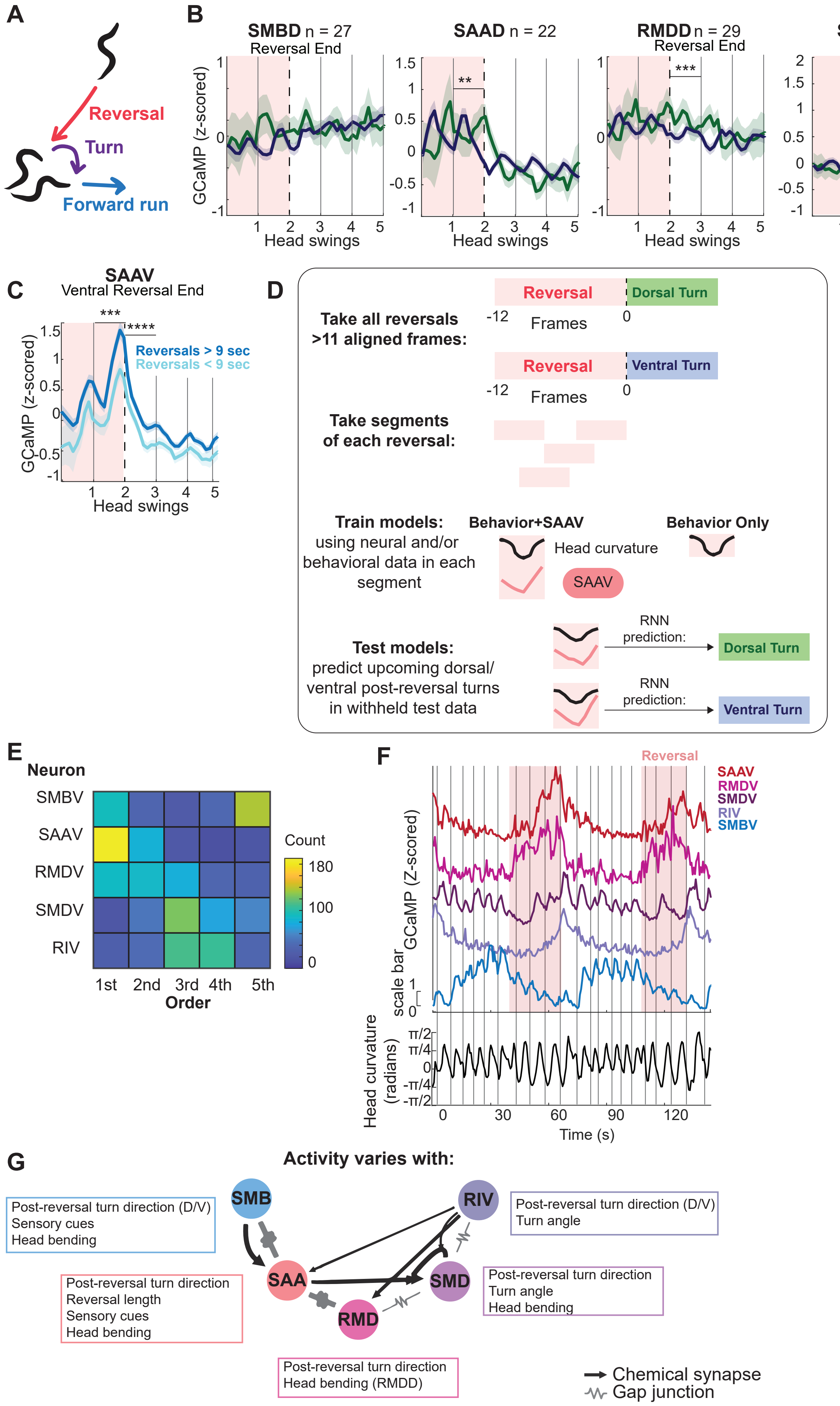

Figure S2.

bioRxiv preprint doi: https://doi.org/10.1101/2024.08.11.607076; this version posted August 11, 2024. The copyright holder for this preprint (which was not certified by peer review) is the author/funder, who has granted bioRxiv a license to display the preprint in perpetuity. It is made available under aCC-BY 4.0 International license.

Supplemental Figure 2, Related to Fig. 2

A) Reorientations are composed of a period of backwards velocity (a reversal), shown in red; then a high angle turn as the animal moves forward, shown in purple; then the forward run, shown in blue.

B) Dorsal counterparts of the neurons shown in Fig. 2B, showing neuron activity across reorientations. Red shading shows reversal; black dashed line is at reversal end. Z-scored activity is aligned to head curvature, data is separated into reversals with dorsal vs ventral post reversal turns. Further alignment description found in Fig. 2B and Methods. n = 115-140 dorsal turn and 415-524 ventral turn reversals. ****$p<.0001$, Wilcoxson's Rank Sum Test with Bonferroni Correction, comparing activity 5 seconds (one head swing) before or after the reversal end. Data are mean ± 95% CI.

C) Average SAAV activity aligned to reversal ends during long and short reorientations with ventral post reversal turns. Red shading shows reversal; black dashed line is at reversal end. Z-scored activity is aligned to head curvature. Data is split by reversal length. SAAV activity is higher in longer reversals (>9 seconds), reflecting that it ramped to a higher activity level during these longer reversals. n = 462 reversals. ****$p<.0001$, Wilcoxon's Rank Sum Test, comparing 5 seconds (one head swing) before or after the reversal end. Data are mean ± 95% CI.

D) Approach used for decoding of upcoming turn direction. Aligned head curvature and SAAV activity (as in Fig. 2B) was taken from all reversals 12 frames or longer (1.5 head swings). Time segments of activity and behavior of length 4 (i.e. 4 frames, which is 2.4 seconds) were then extracted from these reversals. These segments were then used to validate, train, and test Recurrent Neural Networks (RNNs) with five-fold cross validation (more information can be found in Methods) to predict the upcoming post-reversal turn direction. We then compared the decoding accuracy of an RNN trained on behavior and SAAV activity to one trained on behavior alone. See Methods for additional details.

E) Order in which each neuron reaches its peak activity across all recordings with these five neuron classes captured (SAAV, RMDV, SMDV, RIV, and SMBV). To determine the activity order, the time at which each neuron's activity is highest during the transition between reversal to turn to forward was quantified (here, we examined all neuron activity from 1.8 seconds before the reversal end to 7.2 seconds after the reversal end). Based on these times, the order in which the neurons were most active in that reorientation was assigned (first, second, etc). n = 190 reorientations.

F) Example dataset with joint activity recordings of single SAAV, RMDV, SMDV, RIV, and SMBV neurons over two reorientations. Red shading shows reversals, Gray lines show when head curvature crosses from dorsal to ventral (positive to negative). Head curvature for the same animal is quantified on the bottom.

G) The connectivity of the head steering neurons, as in Fig. 2G, here annotated with the behavioral and sensory features that influence each neuron's activity.

bioRxiv preprint doi: https://doi.org/10.1101/2024.08.11.607076; this version posted August 11, 2024. The copyright holder for this preprint (which was not certified by peer review) is the author/funder, who has granted bioRxiv a license to display the preprint in perpetuity. It is made available under aCC-BY 4.0 International license.

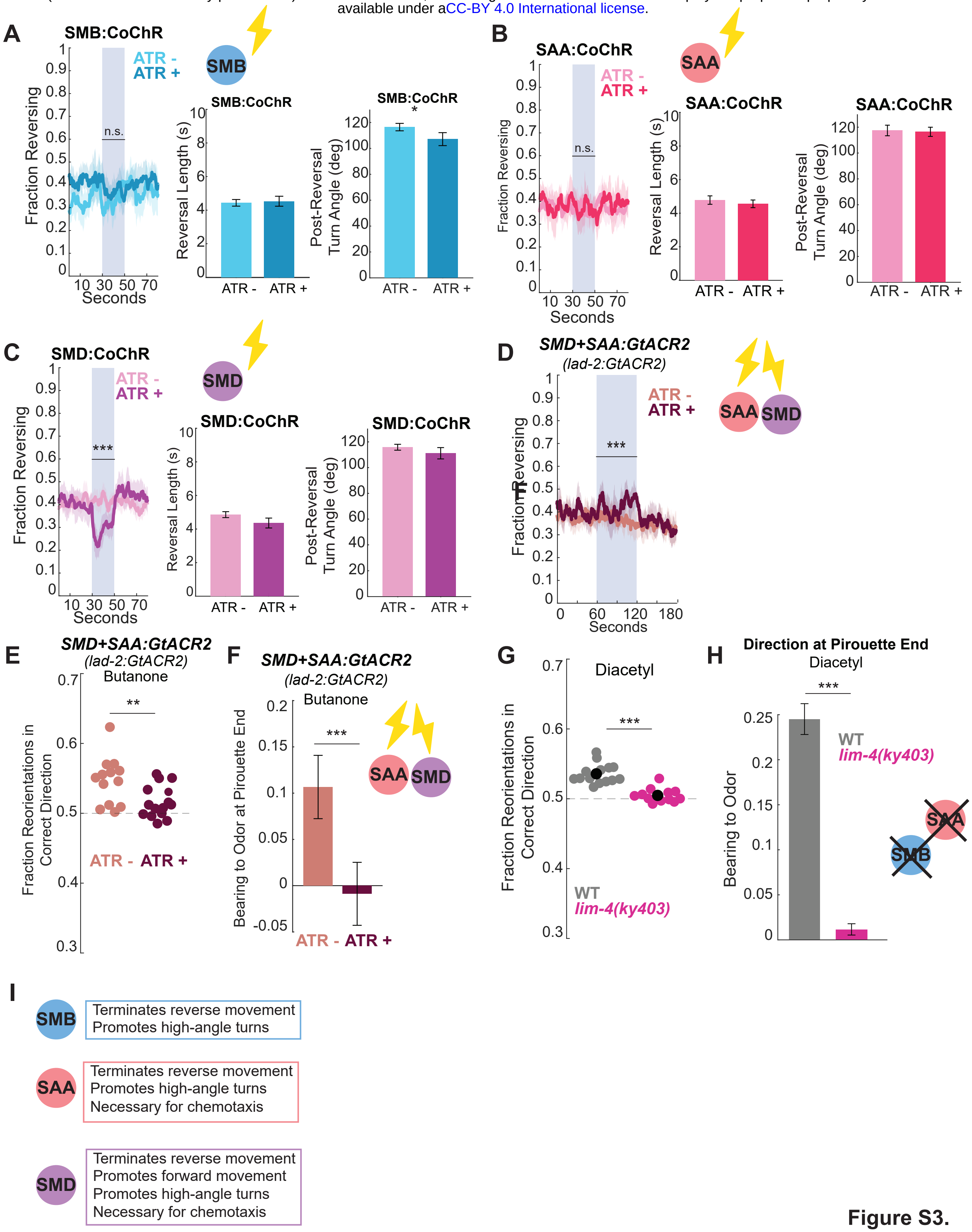

Figure S3.

bioRxiv preprint doi: https://doi.org/10.1101/2024.08.11.607076; this version posted August 11, 2024. The copyright holder for this preprint (which was not certified by peer review) is the author/funder, who has granted bioRxiv a license to display the preprint in perpetuity. It is made available under aCC-BY 4.0 International license.

Supplemental Figure 3, Related to Fig. 3

A-C) Behavioral effects of optogenetically activating the SMBs, SAAs, or SMDs. Cell specific promoters were used to express the excitatory optogenetic CoChR channel in each cell class. SMB is *flp-12(short fragment)::CoChR-sl2-GFP*; SAA is intersectional promoter with *lad-2::cre + unc-42::inv(CoChR-sl2-GFP);* SMD is intersectional promoter with *lad-2::cre + fkh-10::inv(CoChR-sl2-GFP).* Cell specificity was validated using co-expressed GFP. From left to right for each neuron: fraction of animals reversing across time, with the blue bar showing 20 second optogenetic activation via blue light. Reversal length and post reversal turn angle during the stimulus were also quantified. n = 13-14 recording plates, 7 optogenetic stimulations per recording. ****p<.0001, Wilcoxon's Rank Sum Test with Bonferroni Correction, calculating the plate average of the fraction animals reversing or reversal variables during the stimulation, comparing these averages with and without ATR within genotype. For all plots, data are mean ± 95% CI.

D) Behavioral effects of optogenetically inhibiting both the SMDs and SAAs (as well as SDQ, PLN, ALN, expression based on[62]). Animals express *lad-2::GtACR2.* Graph shows fraction of animals reversing across time, with the blue bar showing 60 second optogenetic inhibition via blue light. n = 13-14 recording plates, 6 optogenetic stimulations per recording. ****p<.0001, Wilcoxon's Rank Sum Test, comparing fraction animals reversing per recording plate with and without ATR within genotype. Data are mean ± 95% CI.

E) Fraction animals making the correct dorsal versus ventral turn in a butanone gradient in *lad-2::GtACR2* animals. *lad-2* is expressed in SAA, SMD, and three other neurons[62]. Only reversals that end during the optogenetic inhibition were included in this analysis. Each dot is one plate with 20-100 animals. ****p<.0001, Wilcoxon's Rank Sum Test. n = 13-14 recordings, each dot shows average value from all reversals that end during any of the six stimulations in a recording.

F) Bearing at the ends of pirouettes during butanone chemotaxis in *lad-2::GtACR2* animals. Only pirouettes that end during the optogenetic inhibition were included in this analysis. ****p<.0001, Wilcoxon's Rank Sum Test comparing mean bearing to odor per stimulus with and without ATR. n = 13-14 recordings. Data are mean ± SEM.

G) Fraction reorientations in the correct dorsal/ventral direction during butanone chemotaxis for *lim-4* mutant vs wild type animals. *lim-4(ky403)* animals are cell fate mutants that results in a cell fate change for the SMB neurons, among other cells[63], and morphological deficits in the SAA neurons[71]. In these animals, an aversive sensory neuron, AWB, takes on the cell fate of the butanone-sensing sensory neuron AWC[71]. Therefore, we used the odor diacetyl, which is sensed by the sensory neuron AWA[15], to test these animals' behavior, as past work has shown that *lim-4* mutants can respond to diacetyl[71]. Wild type data here are also shown in Fig. S1E. n = 12-14 recording plates. ****p<.0001, Wilcoxon's Rank Sum Test. Black dots show data mean.

H) Bearing to odor at pirouette ends during butanone chemotaxis for SAA genetic ablation vs wild type animals. n = 12-14 recording plates. ****p<.0001, Wilcoxon's Rank Sum Test. Data shows mean ± SEM.

bioRxiv preprint doi: https://doi.org/10.1101/2024.08.11.607076; this version posted August 11, 2024. The copyright holder for this preprint (which was not certified by peer review) is the author/funder, who has granted bioRxiv a license to display the preprint in perpetuity. It is made available under aCC-BY 4.0 International license.

I) Summary of each cell's functional role, as determined by optogenetic and cell silencing experiments shown in Fig. 3 and S3.

bioRxiv preprint doi: https://doi.org/10.1101/2024.08.11.607076; this version posted August 11, 2024. The copyright holder for this preprint (which was not certified by peer review) is the author/funder, who has granted bioRxiv a license to display the preprint in perpetuity. It is made available under aCC-BY 4.0 International license.

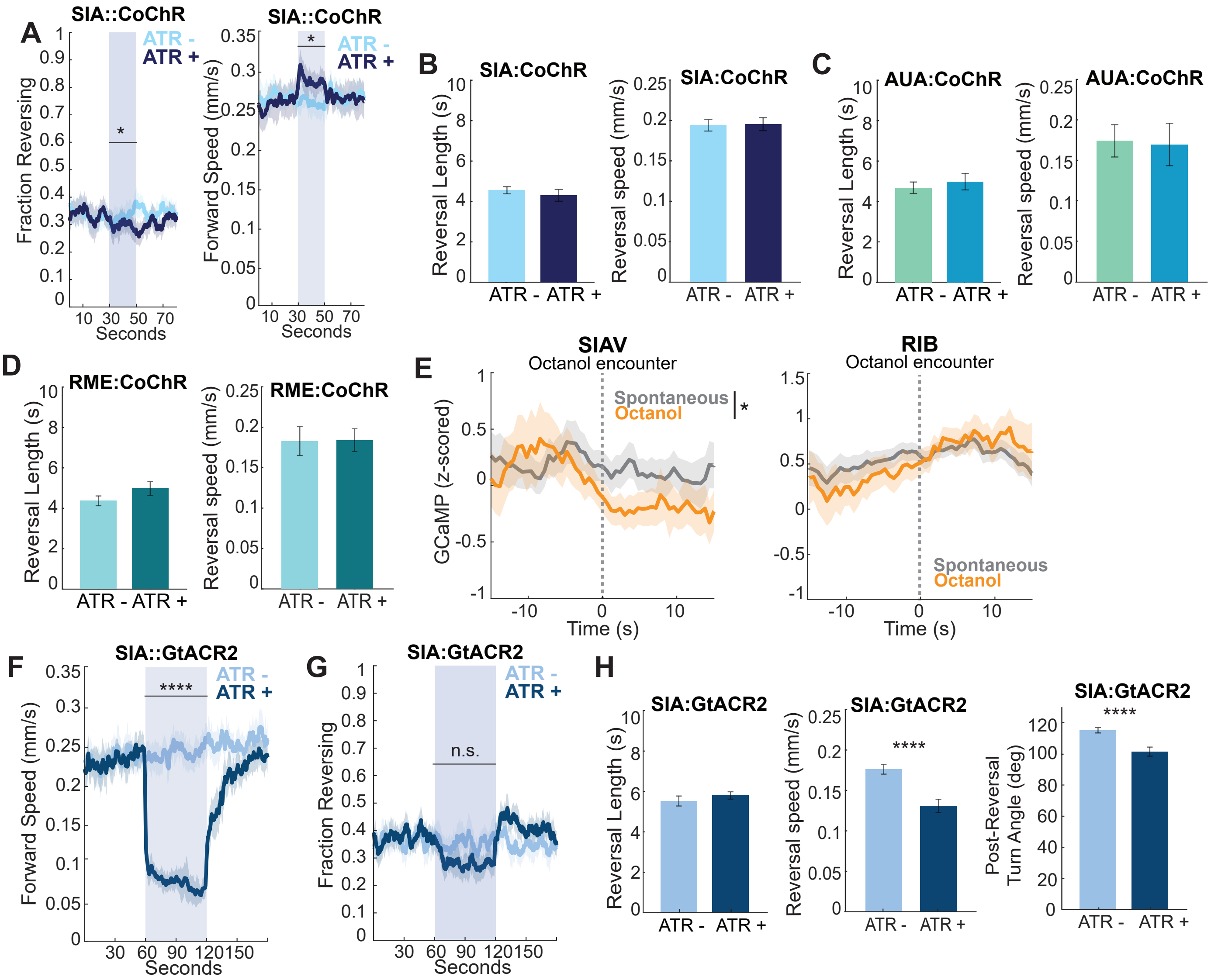

Figure S4.

bioRxiv preprint doi: https://doi.org/10.1101/2024.08.11.607076; this version posted August 11, 2024. The copyright holder for this preprint (which was not certified by peer review) is the author/funder, who has granted bioRxiv a license to display the preprint in perpetuity. It is made available under aCC-BY 4.0 International license.

Supplemental Figure 4, Related to Fig. 4

A) Fraction reversing (left) and forward speed (right) during optogenetic activation of SIA. n = 14-15 recordings, 7 optogenetic stimulations per recording. ****p<.0001, Wilcoxon's Rank Sum Test, comparing average reversal rate or speed with and without ATR. Data are mean ± 95% CI.

B-D) Effects on reversal length and speed during optogenetic activation of SIA, AUA, or RME using the blue light activated CoChR opsin. n = 9-15 recording plates. Wilcoxon's Rank Sum Test with Bonferroni Correction, comparing average behavior values during stimulation for each recording (none of these comparisons are significant). Data are mean ± 95% CI.

E) SIAV and RIB activity as animals encounter the aversive octanol barrier is shown in orange. The gray line shows this neuron's activity during similar length epochs of spontaneous forward movement, to control for how these cell's activity change with the animal's locomotion (for example, consider Fig. 4A). The vertical gray dashed line shows the moment of octanol encounter. *p<.05, Wilcoxon's Rank Sum Test with Bonferroni Correction comparing average activity on octanol and during spontaneous movement, data are mean ± 95% CI.

F) Forward speed during optogenetic inhibition of SIA. n = 15 recording plates per condition, 6 optogenetic stimulations per recording. ****p<.0001, Wilcoxon's Rank Sum Test, comparing average speed with and without ATR. Data are mean ± 95% CI.

G) Percent of animals reversing during optogenetic inhibition of SIA. n = 15 recording plates per condition, 6 optogenetic stimulations per recording. *p<.05, Wilcoxon's Rank Sum Test, comparing reversal rate with or without ATR. Data are mean ± 95% CI.

H) From left to right, effects on reversal length, speed, and post reversal turn angle during optogenetic inhibition of SIA. n = 15 recording plates per condition. ****p<.0001, Wilcoxon's Rank Sum Test with Bonferroni Correction, comparing average values during stimulation for each recording. Data are mean ± 95% CI.

bioRxiv preprint doi: https://doi.org/10.1101/2024.08.11.607076; this version posted August 11, 2024. The copyright holder for this preprint (which was not certified by peer review) is the author/funder, who has granted bioRxiv a license to display the preprint in perpetuity. It is made available under aCC-BY 4.0 International license.

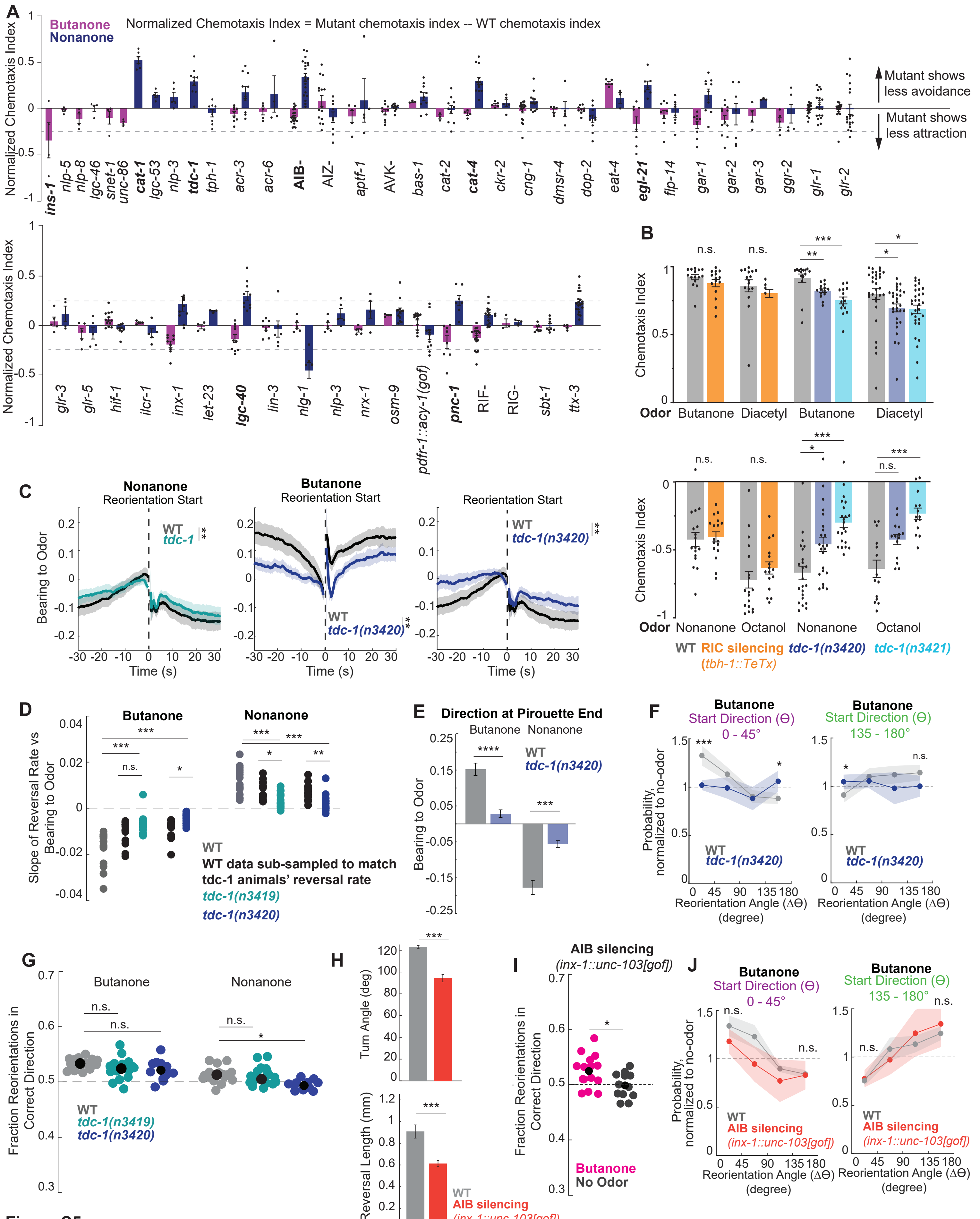

Figure S5.

bioRxiv preprint doi: https://doi.org/10.1101/2024.08.11.607076; this version posted August 11, 2024. The copyright holder for this preprint (which was not certified by peer review) is the author/funder, who has granted bioRxiv a license to display the preprint in perpetuity. It is made available under aCC-BY 4.0 International license.

Supplemental Figure 5, Related to Fig. 5

A) Chemotaxis screen, examining responses to the attractive odor butanone (shown in pink) and the aversive odor nonanone (shown in blue). Chemotaxis indices are normalized to wild type controls run on the same day (that is, the average wild type chemotaxis index for that odor from that day is subtracted from the chemotaxis index from each mutant plate run the same day). Therefore, if mutant strains have a chemotaxis deficit compared to wild type animals, their normalized chemotaxis index will positive for nonanone, and it will be negative for butanone. In the x-axis labels, neuron silencing lines are in capital letters, and endogenous mutations are in italics. Alleles and strains used are listed in the Key Resources Table. Strains where the difference between mutant and wild type chemotaxis is > 0.25 are in bold. n = 3-21 plates over 1+ days with 50-200 animals per plate. Note that not every mutant was tested to both odors.

B) Chemotaxis of wild type and RIC silenced animals (*tbh-1::TeTx*) as well as separate wild type controls and two other alleles of *tdc-1* (*n3420* and *n3421*) to the attractive odors butanone and diacetyl and the aversive odors nonanone and octanol. Chemotaxis index is calculated as (# animals at odor - # animals at ethanol (control)) / (total # of animals). n = 14-31 plates over 3+ days with 50-200 animals per plate. ****p<.0001, Mann Whitney U Test with Bonferroni Correction.

C) Average bearing to odor aligned to reorientation start times, during butanone or nonanone chemotaxis. The dashed line shows reversal start and end. **p<.01, Wilcoxon's Rank Sum Test with Bonferroni Correction comparing the pre-reversal slopes of bearing over time. n = 16-18 recording plates. Data are mean ± 95% CI.

D) Relationship between bearing to odor and reorientation rates in WT and *tdc-1* animals. As in Fig. S1C, this was quantified as the slope of the reversal start vs bearing to odor plot for each recording. In this case, because *tdc-1* animals are less likely to reverse, we wanted to perform a control analysis to examine how a reduced reversal rate would impact these results. Therefore, we randomly removed reversals from wild type data so that they reversed at the same rate as the comparison *tdc-1* genotype (shown in black). Each dot is a single recording. n = 16-18 recording plates. ****p<.0001, Wilcoxon's Rank Sum Test with Bonferroni Correction.

E) Bearing to odor at the ends of pirouettes during butanone or nonanone chemotaxis. ****p<.0001, Wilcoxon's Rank Sum Test with Bonferroni correction. n = 16-18 recording plates. Data are mean ± SEM.

F) Change in direction ($\Delta\Theta$) executed by wild type or *tdc-1(n3420)* animals that start with a small (left, purple) or large (right, green) angle direction to the odor (Θ), normalized to no odor controls. Note that *tdc-1* (blue) does not modulate its turn amplitudes as much as WT animals do. ***p<.001, Wilcoxon's Rank Sum Test with Bonferroni Correction. n = 16-18 recording plates. Data show mean ± 95% CI.

G) Fraction of reorientations that turn the animal in the correct dorsal or ventral direction, comparing wild type, *tdc-1(n3419)*, and *tdc-1(n3420)*. Note that although wild type animals and *tdc-1* animals are not significantly different, *tdc-1* animals do not show a difference in the fraction of correct turns when comparing their own spontaneous and nonanone reorientations (see Fig. 5L). Each dot is one plate with 20-100 animals. *p<.05,

bioRxiv preprint doi: https://doi.org/10.1101/2024.08.11.607076; this version posted August 11, 2024. The copyright holder for this preprint (which was not certified by peer review) is the author/funder, who has granted bioRxiv a license to display the preprint in perpetuity. It is made available under aCC-BY 4.0 International license.

Wilcoxon's Rank Sum Test with Bonferroni Correction. n = 16-18 recordings. Black dots show data mean.

H) Reversals are shorter and smaller angle in AIB silenced animals. AIB silencing is *inx-1::unc-103(gof).* Upper graph compares the absolute value of post-reversal turn angle in wild type and AIB silenced animals, lower compares reversal length. ***p<.001, Wilcoxon's Rank Sum Test with Bonferroni Correction. n = 12-13 recording plates. Data show mean ± 95% CI.

I) Fraction of reorientations that turn the animal in the correct dorsal or ventral direction, comparing AIB silencing *(inx-1::unc-103[gof])* animals in a butanone gradient to no odor movement of the same genotype. *p<.05, Wilcoxon's Rank Sum Test. n = 12-15 recording plates. Black dots show data mean.

J) Change in direction ($\Delta\Theta$) executed by wild type or AIB silencing *(inx-1::unc-103[gof])* animals that start with a small (left, purple) or large (right, green) angle direction to the odor (Θ), normalized to no odor controls. n.s,. p>0.05, Wilcoxon's Rank Sum Test with Bonferroni Correction. n = 12-15 recording plates. Data show mean ± 95% CI.

bioRxiv preprint doi: https://doi.org/10.1101/2024.08.11.607076; this version posted August 11, 2024. The copyright holder for this preprint (which was not certified by peer review) is the author/funder, who has granted bioRxiv a license to display the preprint in perpetuity. It is made available under aCC-BY 4.0 International license.

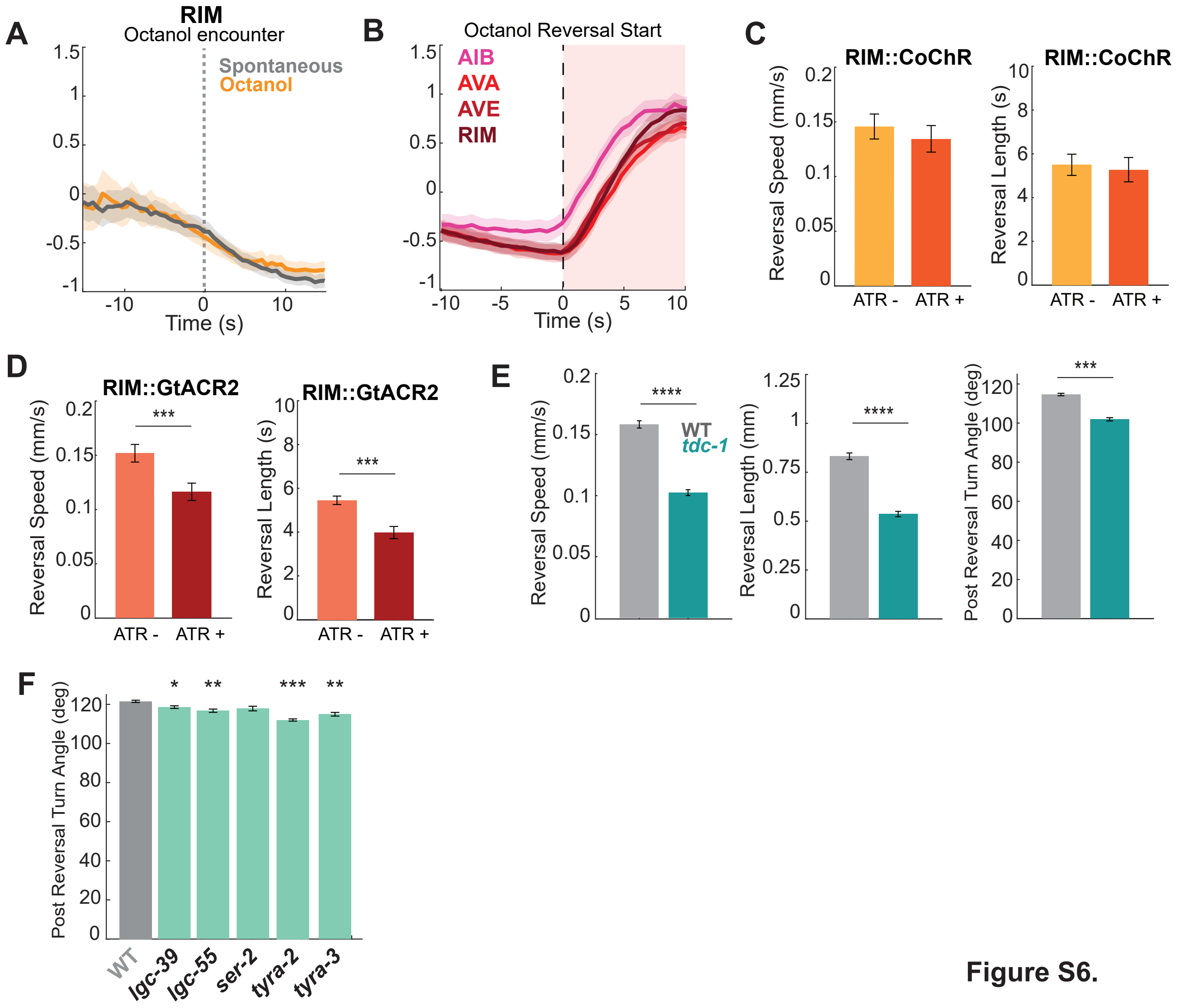

Figure S6.

bioRxiv preprint doi: https://doi.org/10.1101/2024.08.11.607076; this version posted August 11, 2024. The copyright holder for this preprint (which was not certified by peer review) is the author/funder, who has granted bioRxiv a license to display the preprint in perpetuity. It is made available under aCC-BY 4.0 International license.

Supplemental Figure 6, Related to Figure 6

A) RIM activity is unaffected when animals move forward on to octanol. Dashed gray line shows octanol encounter. Spontaneous forward movement epochs are sampled to be a similar length of forward movement as octanol encounters, to allow a comparison of RIM activity during spontaneous forward movement versus forward movement onto octanol. Wilcoxon's Rank Sum Test compares spontaneous and octanol activity (comparison is not significant). Data are mean ± 95% CI.

B) Reversal neuron activity during octanol-triggered reversals (defined as any reversals that begin with the animal's head on octanol). Dashed line shows reversal start, red shading shows the reversal. Data are mean ± 95% CI.

C) Reversal length and speed during optogenetic RIM activation or during spontaneous reversals on no-ATR plates. n = 12-15 recording plates. Wilcoxon's Rank Sum Test with Bonferroni Correction (comparison is not significant). Data are mean ± 95% CI.

D) Reversal length and speed during optogenetic RIM inhibition or during spontaneous reversals on no-ATR plates. n = 11-14 recording plates. ***p<.001, Wilcoxon's Rank Sum Test with Bonferroni Correction. Data are mean ± 95% CI.

E) Reversal speed, length, and post reversal turn angle for wild type and *tdc-1(n3419)* animals. Animals are off food without odor. n = 18 recordings per genotype. ****p<.0001, Wilcoxon's Rank Sum Test with Bonferroni Correction. Data are mean ± 95% CI.

F) Post reversal turn angle for wild type animals and animals lacking each of the five known tyramine receptors. Animals were off food without odor. n = 10 recording plates per genotype. ***p<.001, Wilcoxon's Rank Sum Test with Bonferroni Correction comparing mutant turn angles to wild type. Data are mean ± 95% CI.

bioRxiv preprint doi: https://doi.org/10.1101/2024.08.11.607076; this version posted August 11, 2024. The copyright holder for this preprint (which was not certified by peer review) is the author/funder, who has granted bioRxiv a license to display the preprint in perpetuity. It is made available under aCC-BY 4.0 International license.

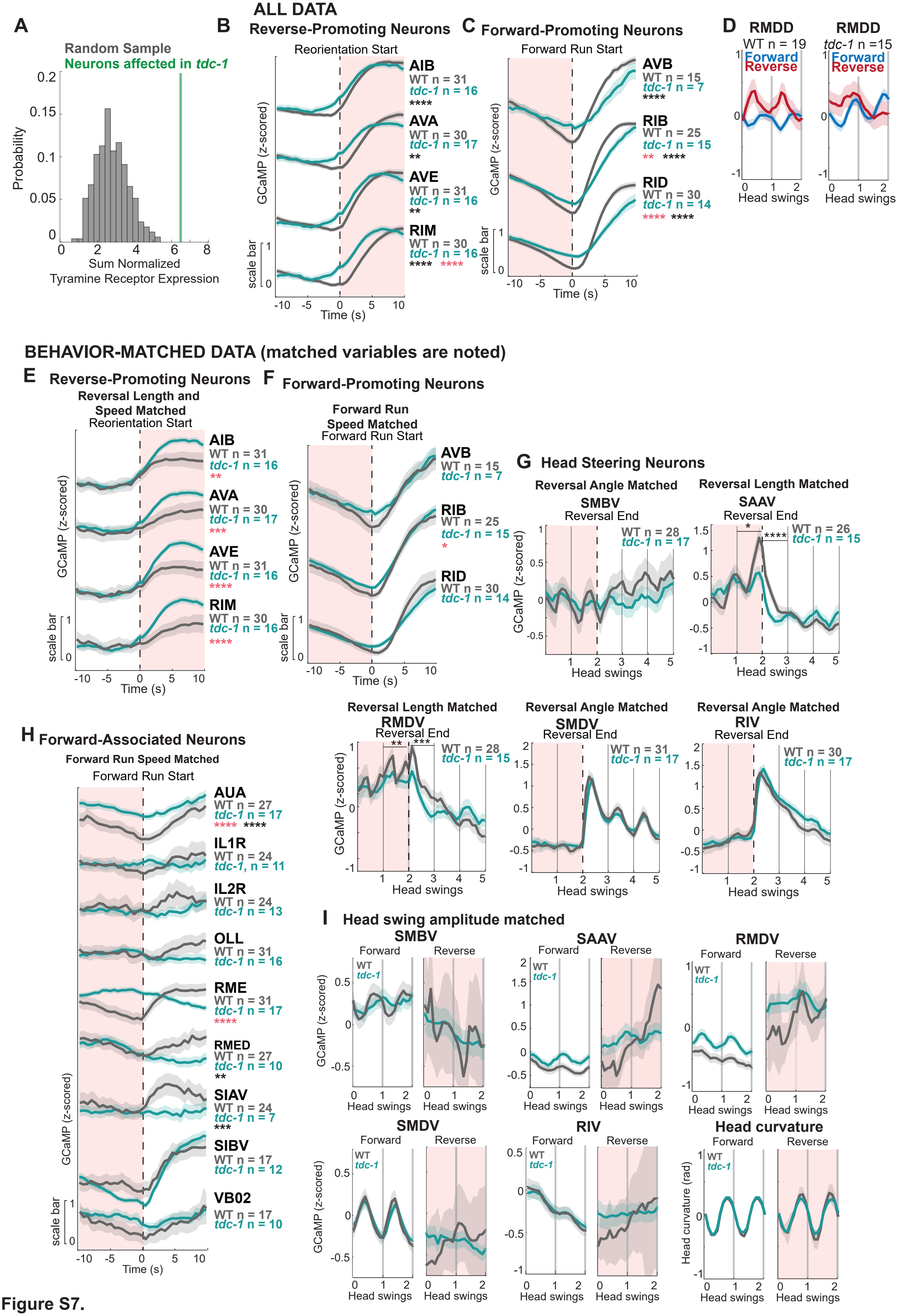

Figure S7.

bioRxiv preprint doi: https://doi.org/10.1101/2024.08.11.607076; this version posted August 11, 2024. The copyright holder for this preprint (which was not certified by peer review) is the author/funder, who has granted bioRxiv a license to display the preprint in perpetuity. It is made available under aCC-BY 4.0 International license.

Supplemental Figure 7, Related to Figure 7

A) Tyramine receptor expression is enriched in neurons that have significantly different encoding of behavior in *tdc-1* animals compared to wild type animals. Gene expression data is from[76], significant changes in encoding are shown and described in Fig. 7A. To compare tyramine receptor expression across receptors and neurons, expression for each neuron was normalized to the maximum Transcripts per Million (TPM) reported in any single neuron for that receptor, resulting in values ranging from 0 (no expression) to 1 (maximum relative expression). For example, *ser-2* is expressed most highly in OLL, at a TPM of 1104. The neuron NSM expresses *ser-2* at expresses 170 TPM, so NSM ser-2 expression is normalized to a value of 0.15. We then compared the sum of expression across all tyramine receptors for all neurons that had altered encoding of behavior in *tdc-1* animals (as reported in Fig. 7A). The green vertical line shows the sum of the normalized tyramine receptor expression for these 9 neurons. The gray distribution shows the distribution of normalized tyramine receptor expression for 500 randomly drawn sets of 9 neurons. This analysis reveals that neurons that show changes in behavior encodings in *tdc-1* animals are significantly more likely to express tyramine receptors than randomly selected neurons, as the real data is at the 99th percentile of the randomly drawn distribution.

B) Average activity of reverse-promoting neurons in all data for wild type and *tdc-1* animals, shown aligned to reorientation starts. Dashed black line shows reversal start; red shading shows the reversal. n = 570-762 reversals. n values on the plot show the number of recordings per genotype with data for a specific neuron. ****p<.0001, Wilcoxon's Rank Sum Test with Bonferroni Correction comparing activity between genotypes both during the run (black stars) and reversal (red stars). Data show mean ± 95% CI.

C) Average activity of forward-promoting neurons in all data from wild type and *tdc-1* animals, shown aligned to forward run starts. Dashed black line shows run start; red shading shows the reversal. n = 218-719 runs. ****p<.0001, Wilcoxon's Rank Sum Test with Bonferroni Correction comparing activity between genotypes both during the run (black stars) and reversal (red stars). Data show mean ± 95% CI.

D) Z-scored RMDD activity aligned to head curvature (as in Fig. 2A) during forward (blue) or reverse movement (red). Left shows wild type data, right shows *tdc-1*. n = 112-447 time intervals of data during either forward or reverse movement. Data are mean ± 95% CI.

As *tdc-1* animals' behavior differs from wild type, and as we know behavior can affect neuron activity (for example, consider how SMDV activity scales with turn angle in Fig. 2C), we wanted to compare neuron activity in wild type versus *tdc-1* animals during similar behaviors. This could allow us to determine whether the relationship between activity and behavior was disrupted in *tdc-1* mutants per se. Therefore, Fig. S7E-I show neuron activity in *tdc-1* and wild type animals during matched behaviors only. This was achieved by taking a subset of the data from either wild type or both wild type and *tdc-1* (indicated in each figure legend) and ensuring that the underlying behaviors were matched for relevant metrics as follows: reversal length, reversal speed, turn angle, or forward run speed. Different variables are controlled for different neurons – the exact variables controlled are determined based on neurons' activities in wild type animals and are specified in the legend and the figure. (For example, SAAV activity changes

bioRxiv preprint doi: https://doi.org/10.1101/2024.08.11.607076; this version posted August 11, 2024. The copyright holder for this preprint (which was not certified by peer review) is the author/funder, who has granted bioRxiv a license to display the preprint in perpetuity. It is made available under aCC-BY 4.0 International license.

based on reversal length in WT animals, so reversal length is matched for WT and *tdc-1* animals when looking at SAAV activity here.)

E) Average activity of reverse-promoting neurons, aligned to reorientation starts, in reversal matched wild type and *tdc-1* animals. Wild type data is limited to activity during reversals with a similar length and speed to *tdc-1* reversals. Dashed black line shows reversal start; red shading shows the reversal. n = 136-641 reversals. ****p<.0001, Wilcoxon's Rank Sum Test with Bonferroni Correction comparing activity between genotypes both during the run (black stars) and reversal (red stars). Data show mean ± 95% CI.

F) Activity of forward-promoting neurons, aligned to forward run starts, in matched wild type and *tdc-1* animals. Wild type and *tdc-1* data are limited to neuron activity during forward runs with similar speeds. Dashed black line shows run start; red shading shows the reversal. n = 77-441 runs. ****p<.0001, Wilcoxon's Rank Sum Test with Bonferroni Correction comparing activity between genotypes both during the run and reversal. Data show mean ± 95% CI.

G) Z-scored neuron activity in neurons of the head steering circuit at reversal endings (dashed black line), after which animals make a turn and resume forward movement. Neural data were aligned to a uniform head curvature frequency to preserve head curvature-associated neuron dynamics (see Fig. 2A and 2B legends). Only reversals followed by ventral turns are shown. Wild type data is limited to reversals of a similar length (SAAV, RMDV) or similar turn angle (SMBV, SMDV, RIV) to *tdc-1* animals. Matching metrics were chosen based on how these neurons respond to these behavior metrics in wild type animals. n = 176-373 reorientations. ****p<.0001, Wilcoxon's Rank Sum Test with Bonferroni Correction, comparing one head swing before or after the reversal end. Data are mean ± 95% CI.

H) Activity of forward-associated neurons, aligned to forward run starts, in matched wild type and *tdc-1* animals. Wild type and *tdc-1* data are limited to forward runs with a similar speed. Dashed black line shows run start; red shading shows the reversal. n = 75-513 runs. ****p<.0001, t-test with Bonferroni Correction comparing activity between genotypes both during the run and reversal. Data show mean ± 95% CI.

I) Z-scored neuron activity aligned to head curvature during forward (left panel) or reverse movement (right, shaded red). Neural activity was aligned to head curvature as in Fig. 2A. As *tdc-1* animals have lower amplitude head curvature (see Fig. 7E), wild type data here is limited to timepoints with similar amplitude head swings to *tdc-1* animals. This criterion resulted in very little wild type data during reversals, hence the large Confidence Intervals. n = 13-411 time windows (each of two head swings). Data are mean ± 95% CI.

bioRxiv preprint doi: https://doi.org/10.1101/2024.08.11.607076; this version posted August 11, 2024. The copyright holder for this preprint (which was not certified by peer review) is the author/funder, who has granted bioRxiv a license to display the preprint in perpetuity. It is made available under aCC-BY 4.0 International license.

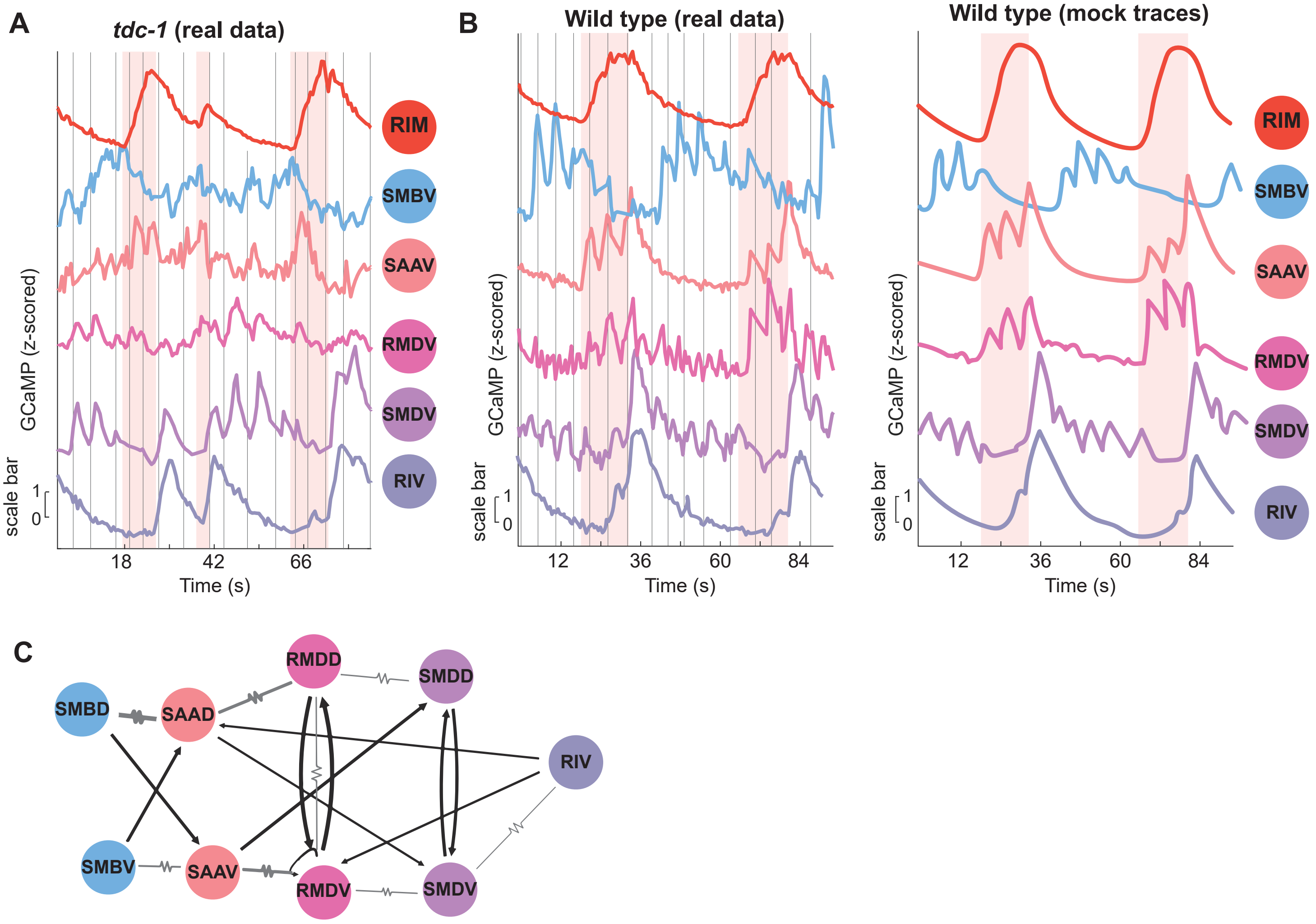

Figure S8.

bioRxiv preprint doi: https://doi.org/10.1101/2024.08.11.607076; this version posted August 11, 2024. The copyright holder for this preprint (which was not certified by peer review) is the author/funder, who has granted bioRxiv a license to display the preprint in perpetuity. It is made available under aCC-BY 4.0 International license.

Supplemental Figure 8, Related to Figure 7

A) Calcium traces of RIM and the neurons of the head steering network in an individual *tdc-1* animal across three reversals (shaded in red). Gray vertical lines show head curvature crossings from dorsal to ventral. These traces can be compared to the same neurons in a single wild type animal (B). Similar to when data is pooled across animals (Fig. 7D-E, S7G,I), responses in SMDV and RIV are largely unaffected in *tdc-1* animals compared to wild type, while SAAV, RMDV, and SMBV activity are dysregulated in a *tdc-1* background.

B) Calcium traces of RIM and the neurons of the head steering network in a wild type animal across two reversals (shaded in red), showing each neuron's stereotyped, sequential responses across each reorientation. Gray vertical lines show head curvature crossings from dorsal to ventral. The left plot shows real traces of each of the neurons, which were recorded simultaneously in the same animal. The right plot shows stylized mock traces for each neuron, which are presented in Fig. 7G as well. Mock traces were drawn based on the actual data to the left.

C) Connectivity of the head steering network, separating out each neuron class into its dorsal/ventral subtypes. This presentation reveals several nuances to the network connectivity; for example, gap junctions connect adjacent "D" class neurons and adjacent "V" class neurons, which could be related to their sequential activation. In addition, SAAV projects to SMDD, while SAAD projects to SMDV. This motif could allow the more active SAA to perhaps suppress the activity of the opposing SMD at the transition between reversals ending and turns beginning. Also of note are the opposing chemical synapses from SMBD to SAAV and SMBV to SAAD, which could allow propagation of sensory-responsive signals between these cell classes based on directional information from the surroundings.
